# Orthopaedic Management of Gout

**DOI:** 10.5435/JAAOSGlobal-D-22-00216

**Published:** 2022-11-08

**Authors:** Anna R. Cohen-Rosenblum, Jason R. Somogyi, Kelly K. Hynes, Myriam E. Guevara

**Affiliations:** From the Department of Orthopaedic Surgery, Louisiana State University Health Sciences Center, New Orleans, LA (Dr. Cohen-Rosenblum); the Texas Orthopedics; Affiliate Faculty, Department of Surgery and Perioperative Care, Dell Medical School, The University of Texas, Austin, TX (Dr. Somogyi); the Department of Orthopaedic Surgery and Rehabilitation, University of Chicago Medicine, Chicago, IL (Dr. Hynes); and the Department of Medicine, Section of Rheumatology, Louisiana State University Health Sciences Center, New Orleans, LA (Dr. Guevara).

## Abstract

Gout is characterized by the deposition of monosodium urate crystals in patients with chronically elevated blood levels of uric acid. It is the most common form of inflammatory arthritis in the United States and is often comorbid with hypertension, obesity, and chronic kidney disease. Initial presentation is usually an acutely warm, swollen joint, most commonly the first metatarsophalangeal joint, but a variety of locations may be affected. The main treatment for gout is medical management of acute inflammation and chronic uric acid levels, but surgical treatment may be indicated in cases of damage to the surrounding soft tissue, concomitant septic arthritis, symptomatic cartilage loss, or neurologic deficits. Based on the literature to date, gout does not seem to independently affect outcomes after total hip, knee, and ankle arthroplasty, but associated comorbidities affecting outcomes in these patients should be considered.

Gout is an inflammatory arthritis characterized by the deposition of monosodium urate (MSU) crystals in patients with chronically elevated uric acid levels.^1^ The term gout comes from the Latin *gutta* (drop), which is thought to relate to the concept of an imbalance of humors flowing into a joint.^2^ This condition affects approximately 4% of the adult population in the United States and has a variety of associated risk factors including certain medications, dietary factors, and medical conditions such as chronic kidney disease, obesity, congestive heart failure, hyperlipidemia, and lead exposure.^1^

Acute manifestations of gout include a painful, swollen joint that can either be mistaken for septic arthritis or concurrently infected,^1^ whereas chronic tophaceous deposits can cause periarticular bony erosions and damage to surrounding nerves and tendons.^3^ Orthopaedic surgeons are likely to encounter both acute and chronic gout over the course of their careers. This article will review the pathophysiology and medical treatment of gout and surgical treatment of gout in the upper and lower extremity and spine.

## Pathophysiology of Gout

The pathophysiology of gout can be divided into four stages: (1) hyperuricemia, (2) MSU crystal deposition without symptoms, (3) MSU crystal deposition with gout flare, and (4) chronic gout with tophi.^1^

Hyperuricemia (>6 mg/dL) occurs from overproduction or underexcretion of uric acid. Overproduction is caused by high cell turnover from conditions like psoriasis or myeloproliferative disorders, inherited enzyme abnormalities in the urate biosynthesis pathway, and high intake of purine-rich foods such as alcohol, red meat, seafood, and high fructose corn syrup. Underexcretion is caused by chronic kidney disease, metabolic syndrome, diuretic use, and other medications and genetic abnormalities affecting urate transport in the kidneys and intestines.^1^

Once hyperuricemia is present, some patients may form uric acid crystals. Peripheral joints with lower temperatures are more conducive to MSU crystal formation.^1,2^ Once intra-articular MSU crystals have formed, they interact with resident macrophages to cause an inflammatory response by activating neutrophils and the release of cytokines.^4^ Over time, MSU crystals are surrounded by granulomas to form a tophus that erodes into periarticular bone.^1^

## Diagnosis

### Clinical

An acute gout flare presents as a painful, swollen joint. Patients may complain of severe throbbing pain at night waking them from sleep and pain with ambulation if the foot and ankle are affected. They may note having had similar symptoms during previous gout flares if the condition is long standing. There may be a recent trigger, such as dehydration, alcohol/purine ingestion, illness, or surgical procedure. On examination, patients may have fever, pain with active and passive range of motion of the affected joint, tenderness to palpation, warmth, and erythema. Chronic tophaceous gout appears as subcutaneous nodules that are normally painless but become painful during a flare. The skin overlying the nodules may erode, revealing chalky tophaceous material. Ulceration over a gouty tophus is a risk factor for superinfection. Gout flares can occur in any joint but occur most commonly in the foot and ankle, specifically the first metatarsophalangeal joint.^1^

If a patient with a painful, swollen joint has a history of gout and similar symptoms from previous flares, a gout flare is likely. In the case of no known previous gout history, atypical symptoms, or other risk factors for infection such as immunocompromise or overlying skin ulceration, gout diagnosis must be confirmed with aspiration to rule out septic arthritis, superinfected gout, or a different crystalline arthropathy such as pseudogout/calcium pyrophosphate dihydrate (CPPD) crystal deposition.^1,5^

### Laboratory Analysis

The benchmark for diagnosis of gout is the appearance of negatively birefringent needle-shaped crystals in synovial fluid or tophaceous material under polarized light microscopy.^1^ This is in contrast to CPPD crystals, which will appear rhomboid shaped and positively birefringent.^6^ In addition to crystal analysis, synovial fluid samples should always be sent for cell count with differential, Gram stain and culture.^7^ Synovial fluid white blood cell (WBC) counts under 50,000 cells/μL (or mm^3^) in the presence of MSU crystals are generally not suspicious for septic arthritis.^3^ Special attention must be paid even in cases of synovial WBC below this level if patients are immunocompromised and unable to mount the immune response of a high synovial fluid WBC count.^7^ In addition, ruptured tophi with overlying skin ulceration should increase suspicion for possible coinfection.^1,7^ Synovial fluid cultures should be followed over time even in patients with low suspicion of infection, and Gram stains have a high positive predictive value but cannot exclude septic arthritis if negative.^5,7^

In addition to aspiration, other laboratory tests can help confirm a diagnosis of suspected gout. C-reactive protein (CRP) is usually increased during a gout flare and can be quite high, although a high CRP alone is nonspecific. In other words, a high CRP alone does not necessarily mean that septic arthritis is present, whereas a CRP under 100 mg/dL in the setting of a lower synovial fluid WBC count and the presence of MSU crystals is generally consistent with gout.^1,5^ Serum WBC may or may not be elevated during a gout flare depending on the amount of systemic inflammation, and therefore, the test is less useful.^1^ Serum urate levels are also less useful as they can be normal during a gout flare, and conversely, many patients with higher serum urate levels do not have gout.^1^ Finally, blood cultures should be ordered in patients with signs of sepsis and if positive increase suspicion for septic arthritis.^5,7^

### Imaging

**Figure 1:**
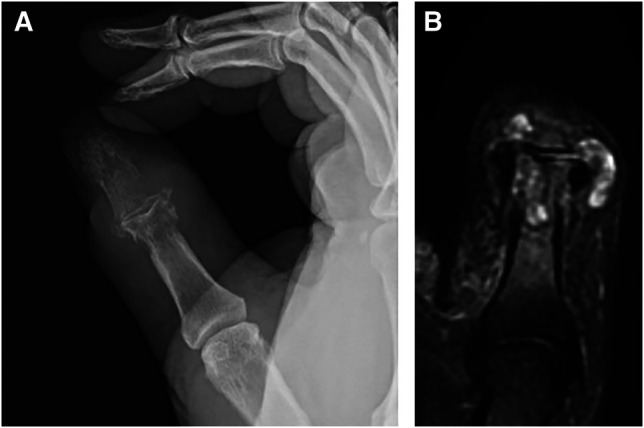
**A**, Radiographic imaging of gout in the thumb (author's clinical photograph). **B**, MRI of gout in the thumb (author's clinical photograph).

In general, diagnosis of gout is achieved by clinical findings and laboratory analysis. There are no specific radiographic findings present in an acute gout flare, whereas chronically affected joints may show periarticular erosions with sclerotic margins.^8^ MRI will also show bony erosions (if present), bone marrow and/or soft-tissue edema, synovial pannus, and gouty tophi in chronic cases.^9^ Figure 1, A and B shows corresponding radiographic and MRI of periarticular erosions and gouty tophi in the thumb. Tophi may appear hypointense or isointense on T1, variable signal on T2, and enhance homogeneously with contrast.^8^ Ultrasonography of gouty arthropathy will show effusions, synovitis, and even MSU crystals themselves. The ultrasonographic double-contour sign refers to irregular hyperechoic enhancement of the articular surface representing MSU crystal deposition that has been described in patients with chronic gout and subcutaneous nodules on physical examination.^8,10^ More recently, dual-energy CT (DECT), an imaging method that differentiates materials “based on their relative absorption of X-rays at different photon energy levels,” has been used to identify patterns of MSU crystal deposition via color coding and can be useful in atypical presentations of gout or monitoring chronic gout over time.^8^

Table 1 provides a brief overview of gout diagnostic criteria.

**Table 1:** Diagnostic Criteria for Gout

Clinical	Laboratory	Imaging
Painful, swollen joint, ± erythema (acute flare) • Most commonly first MTP Ulceration/soft-tissue mass (chronic tophus) Fever	Serum: • Elevated CRP and urate Synovial fluid: • Negatively birefringent needle-shaped crystals • Cell count <50,000 WBCs/μL	Radiography: • Acute: no abnormalities (acute flare) • Chronic: periarticular erosions with sclerotic margins Ultrasonography or DECT findings of MSU crystal deposition

CRP = C-reactive protein, DECT = dual-energy CT, MTP = metatarsophalangeal, MSU = monosodium urate, WBC = white blood cell

## Treatment

### Medical Treatment

The goal of medical treatment of gout is acutely to control inflammation from flares and chronically to lower uric acid levels. Gout flares are usually treated with NSAIDs (naproxen, indomethacin, or selective cyclooxygenase 2 inhibitors), colchicine, and oral or intra-articular corticosteroids, either individually or in combination.^1,2^ Patients whose gout flares are refractory to these traditional treatments or have medical contraindications may be considered for monoclonal antibodies against IL1 or adrenocorticotropic hormone.^11^ Table 2 provides recommendations for treatment of acute gout flares.

**Table 2:** Medications for Gout Flares

Drugs	Use	Cautions	Comments
**NSAIDs** Naproxen 500 mg PO BID Indomethacin 50 mg PO TID	Within 24 hr of a gout flare for 4-10 d	Elderly, renal insufficiency, heart failure, peptic ulcer disease, liver disease, and concurrent anticoagulants	First-line therapy
**COX 2 inhibitors** Celecoxib single dose of 800 mg followed by 400 mg 12 hr later, then 200 mg PO twice daily	Within 24 hr of a gout flare for 7-10 d	History of coronary artery bypass graft surgery (contraindicated)	Off-label use for gout, but equivalent relief to indomethacin in some studies
**Colchicine** 1.8 mg total for day 1, then 0.6 mg QD or BID as tolerated	Within the first 12-36 hr of a gout flare for maximum effectiveness, can be an alternative to NSAIDs or steroids	Kidney or liver disease, on a statin, cytochrome P450 3A4 inhibitor, (verapamil and clarithromycin) or p-glycoprotein inhibitors (ciclosporin) The most common adverse effect is diarrhea and abdominal cramping	Reduce dose in elderly patients and avoid IV use
**Corticosteroids** Prednisone or prednisolone. Use 30-40 mg once daily or in two divided doses	Use until resolution of flare begins, then taper dose over 10-21 d	Common adverse effects: mood changes, hyperglycemia, increased blood pressure, and fluid retention	Consider intra-articular corticosteroid injection with triamcinolone or methylprednisolone, but avoid in patients with suspicion for concurrent septic arthritis
**IL 1 inhibitors** Canakinumab 150 mg single dose Anakinra 100 mg QD	Reserved for patients who have failed or have contraindications to options above	Anakinra generally preferred given shorter half-life and lower cost	Most expensive treatment option

COX 2: cyclooxygenase 2

The first-line treatment for lowering uric acid is with xanthine oxidase inhibitors, which stop the synthesis of uric acid from hypoxanthine and include allopurinol and febuxostat. Next, consideration is given to medications that promote uric acid excretion (uricosuric) such as probenecid and those that promote uric acid degradation such as pegloticase, an intravenously administered recombinant uricase. Newer therapeutics include arhalofenate, which has been shown to have both uricosuric and anti-inflammatory effects in clinical trials, and oral recombinant uricases.^1,12^

Finally, lifestyle modifications such as weight loss and limiting the intake of purine-rich foods should also be recommended in addition to medications for decreasing inflammation and uric acid levels.^1^

### Surgical Treatment

Surgical treatment for gout is generally reserved for cases of damage to the surrounding soft tissue, concomitant septic arthritis, symptomatic cartilage loss, or neurologic deficits. The following sections will describe surgical treatment for gout in the upper and lower extremities and spine and outcomes in patients with gout after total hip, knee, and ankle arthroplasty.

## Upper Extremity

### Shoulder/Elbow

Clinical manifestations of gout in the shoulder and elbow are rare, possibly due to increased solubility of urate in warmer joint compared with the cooler, peripheral joints of the hand and foot. Case reports of gout involving the rotator cuff describe patients with examination findings resembling calcific tendinitis and rotator cuff impingement that were treated surgically with open or arthroscopic subacromial decompression and débridement and found on laboratory analysis to have tophaceous gout.^13^ Gouty tophi have also been described in the olecranon bursa and may be treated with surgical excision in cases of severe limitation of function.^14^

### Wrist

Patients with gout flares in the wrist usually present with pain, erythema, and edema. Wrist aspiration is important to differentiate from pseudogout, cellulitis, hematoma, arthritic flare, or septic arthritis.^15^ Chronic gout in the wrist may lead to peritendinous tophi, lytic lesions in the carpal bones, and crystal deposition causing intercarpal ligament pathology, including scapholunate dissociation.^16^ Arthroscopic intervention in patients with gout and scapholunate ligament has shown diffuse radiocarpal and midcarpal synovitis crystalline deposits, cartilage wear, and scapholunate and lunotriquetral ligament disruption.^17^

Surgical intervention of gout in the wrist is reserved for cases with persistent symptoms after medical management. Wrist arthroscopy can be used for synovectomy and evaluation of intercarpal ligamentous integrity and articular surfaces. Ligamentous reconstruction can proceed if the articular surfaces are preserved. If arthritis is present, standard management with salvage procedures such as proximal row carpectomy, intercarpal fusion, or wrist arthrodesis may be indicated. Although reports of concomitant gout and septic arthritis of the wrist are rare, arthroscopic treatment could be considered as an alternative to open synovectomy; a study of 40 septic wrists found that arthroscopic irrigation and débridement had a similarly effective rate of infection treatment with decreased length of stay compared with open treatment.^18^ It is imperative that uric acid control continue beyond surgical intervention.^19^

Peritendinous gouty tophi can present in the flexor tendons at the carpal tunnel or as dorsal wrist tenosynovitis, occasionally leading to tendon rupture. Extensor pollicis longus rupture due to infiltrating tophi has been reported and was managed with open débridement and extensor indicis proprius to extensor pollicis longus transfers,^20^ whereas Pai and Tseng reported a case of acute carpal tunnel syndrome and imaging with calcific densities in the wrist, treated with open carpal tunnel release and flexor synovectomy with histologically confirmed tophi infiltrating the flexor tendons.^21^ Finally, acute gout may occur after carpal tunnel release in patients with a history of gout flares in the lower extremity who had no evidence of tophaceous material in the carpal tunnel at the time of surgery.^22^ These patients may be managed medically to control inflammation and uric acid levels after ruling out infection.

### Hand

Gout flares and tophi in the hand tend to occur more often in the distal interphalangeal (DIP) joint, which may be related to cooler temperatures and age-related arthritis changes predisposing to uric acid crystal formation. DIP gouty tophi can often be treated by aspiration and manual expression of tophaceous material. Arthrodesis may be indicated for painful DIP joints with joint destruction. Proximal interphalangeal (PIP) joints may also be affected by gout, leading to contractures or extensor lag. Following the failure of medical management, gout in the PIP can be treated surgically based on the patient's functional needs, ranging from simple synovectomy and removal of tophi to PIP arthrodesis. In the rare case of metacarpophalangeal joints with painful persistent gouty tophi, surgical excision can be performed, but care must be taken to ensure extensor tendon continuity. If integrity has been lost, the distal stump of the tendon should be sutured to an adjacent intact extensor tendon.^19^

### Digital Flexor Tenosynovitis

**Figure 2:**
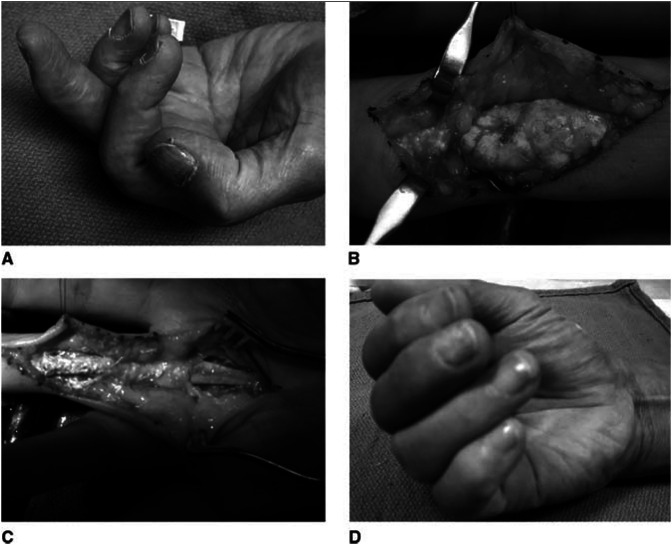
Gouty tophi surrounding the flexor tendon.^19^ **A**, Clinical appearance of a middle finger with chronic tophi impeding flexor tendon excursion. **B**, The tip of the finger is to the left. The tophus is seen distending the distal half of the A2 pulley along with A3, C2, and the proximal edge of the A4 pulley. **C**, After débridement of the tophus and careful maintenance of the A2 and A4 pulleys, the tendons displayed marked improvement in excursion. **D**, Note improved (but not full) composite flexion postoperatively.

Similar to infectious flexor tenosynovitis, gout in the flexor tendons of the hand can present as a painful swollen digit. Surgical intervention is indicated when there is impaired of function of the flexor tendon due to concern for rupture or impending rupture. A high suspicion for gouty flexor tenosynovitis should be considered in patients with a history of gout, elevated uric acid level, and a flexor tendon nodule (which can also be confused with Dupuytren disease). Weniger et al.^23^ presented three cases of gouty tenosynovitis treated surgically with exploration, tenosynovectomy, and débridement of tophi. Figure 2 shows intraoperative photographs reported by Fitzgerald et al.^19^ of chronic tophi in the flexor tendon. In cases of significant tophaceous tendon infiltration with poor tissue for repair, a tendon transfer or bridge graft may be used to restore flexor tendon function.

## Lower Extremity

### Hip

Gout in the hip is quite rare and per some reports less symptomatic than flares in smaller joints.^24^ Chronic periarticular erosions accompanied by symptomatic degenerative changes refractory to nonsurgical management may be treated by primary total hip arthroplasty (THA). A 2019 case report by Huang et al. of THA in the setting of chronic gout noted acetabular cysts (which the authors ascribed to gouty erosion) requiring bone graft, necrotic tissue, and multiple granules diagnosed as gouty tophi based on histologic analysis. They treated the patient with xanthine oxide inhibitors and 48 hours of intravenous antibiotics in the perioperative period.^24^ A case report by Jeon et al. describes a pathologic femoral neck fracture in the setting chronic tophaceous gout in a 48-year-old man who was treated with THA. The authors note that they had originally planned on fracture fixation, but changed to recommending THA when considering the patient's significant preexisting functional limitations due to gout in his hands and feet. They describe removing a chalky and white paste-like mass from the hip joint and recommend consideration of prophylactic proximal femur fixation in the setting of chronic gout with significant erosive changes, using the Mirel criteria.^25^

### Knee

The knee is a far more common site of gout involvement than the hip and also is a frequent site of CPPD crystal deposition disease.^6^ Reviews of concomitant septic arthritis and crystal arthropathy have found that the knee is the most commonly affected joint for this condition^5,7^; therefore, it is important to consider the possibility of infection when treating gout in this location and always send synovial fluid for culture. Concomitant gouty and septic arthritis should be treated with urgent arthroscopic or open surgical débridement and culture-directed antibiotics. Studies of native knee septic arthritis treatment have found that arthroscopic treatment resulted in more successful eradication of infection and improved range of motion^26^ and decreased length of stay and surgical time,^27^ so this may be a consideration when determining treatment.

Although less common than an intra-articular flare with or without septic arthritis, gout in the knee can also damage periarticular structures. There are two case reports in the literature describing bilateral spontaneous quadriceps tendon ruptures in patients with chronic gout, both treated with intratendinous suture repair and uric acid control.^28^ Gouty tophi have been described in the patellar tendon and inside the patella itself, with the recommended treatment being uric acid–lowering therapies to prevent pathologic tendon rupture or pathologic patellar fracture.^9^

### Outcomes After Total Hip Arthroplasty/TKA

The effects of gout on outcomes after THA and total knee arthroplasty (TKA) are not entirely clear. A 2016 retrospective case-control study matching 482 primary THA and TKA patients with gout to patients without gout at a single institution found that the gout group had a higher risk for wound healing problems and renal complications. The authors conclude that patients with gout undergoing elective THA or TKA should have preoperative optimization of uric acid and renal function tests and appropriate postoperative fluid management and prompt treatment of wound complications, noting that these adverse outcomes may also be related to gout's associated comorbidities.^29^

A 2019 National Inpatient Sample database cohort study of over four million primary THA patients matched those with and without a gout diagnosis, finding an overall gout prevalence of 2.5% and no association between gout and postoperative prosthetic joint infection (PJI) or revision.^30^ It is important to note that this study was not able to clarify whether gout was affecting the hip itself as opposed to a comorbidity affecting other locations. A 2021 review of Medicare data from over 1 million primary TKA patients found a higher overall gout prevalence of 5.7%, and similarly did not find any difference in PJI or revision, concluding that gout is not an independent risk factor for adverse outcomes after TKA but that surgeons should be aware that patients with gout often have other risk factors such as obesity and renal disease and counsel accordingly.^31^

Finally, periprosthetic gout flares have been described following TKA and raise the controversial question as to whether these cases should be treated with urgent surgical irrigation and débridement and possible polyethylene exchange or medically to decrease inflammation and lower uric acid levels.^32^ In cases of suspected acute postoperative or acute hematogenous PJI, urgent surgical treatment is recommended; however, if the cause of acute periprosthetic swelling, pain, and effusion with elevated synovial WBC is a gout flare, this could, in theory, be treated with medical management and monitoring synovial fluid cultures. The authors of this case report note that while “aseptic gouty arthritis can be successfully managed with gout medications alone, this is not the routine treatment”.^32^

### Foot/Ankle

The foot and ankle is a very common area for gouty involvement, with the first metatarsophalangeal joint (first MTPJ) being the most common site at 85% to 90% of all cases.^33^ Surgical intervention is indicated for severe disease that has resulted in joint destruction, active concomitant infection, deformity, and/or gait dysfunction.

### Tibiotalar Joint

Although the tibiotalar joint is a common location for gout flares, epidemiologic studies have found that very few patients with severe tibiotalar arthritis have a history of gout.^34^ Gout flares with concomitant septic arthritis should be managed with surgical débridement, whereas patients with severe joint destruction may be managed with ankle fusion or total ankle arthroplasty (TAA) similar to primary osteoarthritis. There is a paucity of literature regarding TAA outcomes in the setting of gout. One small study found that patients with gout and TAA were overall satisfied with their functional outcomes with a single incidence of aseptic loosening.^35^ Although TAA can be successful in the setting of gouty arthritis, larger studies need to be conducted to look at long-term outcomes.

### Hallux

Patients presenting with first MTPJ involvement with gout may have hallux valgus and osteoarthritis and often report restricted range of motion of the first MTPJ. Joint destructive features and hallux valgus deformity are often concurrent in this patient population^36^ The primary indications for surgical treatment of the first MTPJ are destructive arthropathy resulting in pain or deformity, a tophus that creates difficulty with shoewear, or superinfection of a gouty tophus.^33^ In cases of a painful tophus without underlying bony pathology, tophus excision alone may be sufficient, but patients should be counseled regarding the risk of wound complications, recurrence, and infection.^37^ If a large soft-tissue defect is anticipated after tophus removal, consideration can be given to soft-tissue rearrangement with skin graft and/or flap coverage.^37^ Amputation may be required if salvage is not possible.

In cases of soft-tissue destruction and resultant hallux valgus deformity or destructive arthritis, an MTPJ fusion may be indicated.^38^ Joint-sparing hallux valgus procedures such as corrective osteotomies with soft-tissue correction, implant arthroplasty procedures, or soft-tissue interposition arthroplasty have specifically been studies in patients with gout. Although hallux valgus can occur in the setting of gout, there is often associated joint arthropathy, which precludes performing joint-sparing procedures. Although interposition arthroplasty has been studied in the hallux rigidus literature, this type of procedure should be considered with caution in the setting of gout, when the joint capsule and surrounding soft tissue are often compromised as well, which could limit the effectiveness of a soft-tissue interposition procedure. No specific techniques in performing a first MTPJ fusion specific to gout have been described in the literature; however, if there is significant bone loss, such as in the setting of prior gout-associated septic joint, a bone block arthrodesis may be required.

If a bone block arthrodesis were to be attempted in the setting of a prior septic first MTPJ, a multidisciplinary approach should be taken including consideration for a two-stage approach with first a cement spacer placement, followed by antibiotic treatment under the supervision of an infectious disease specialist. Kirschner wires or an external fixator may be used to stabilize the cement spacer if necessary.^39^ Only when the infection is cleared, should implantation of a bone graft and internal fixation be performed.

**Figure 3:**
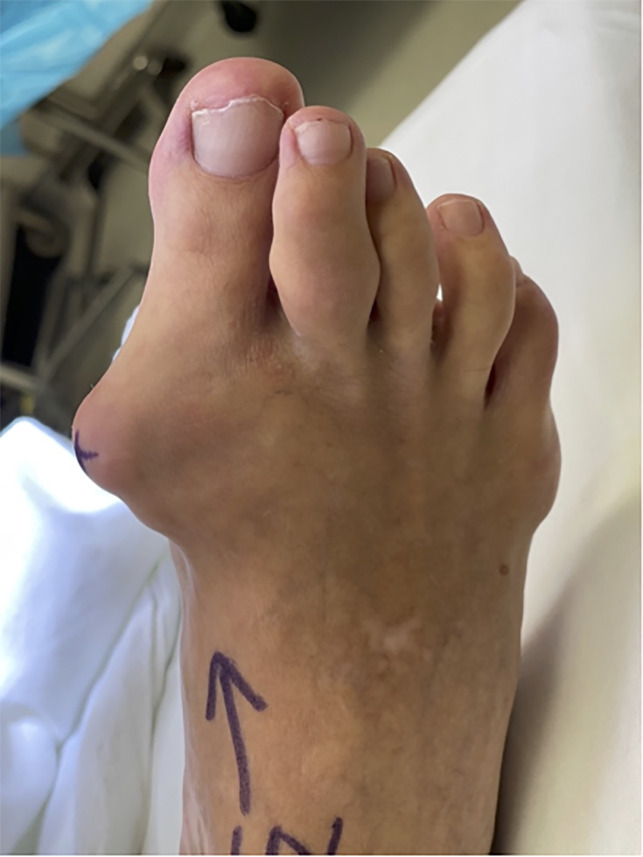
Gouty tophus associated with hallux valgus deformity and painful tophus in the second toe (author’s clinical photograph).

**Figure 4:**
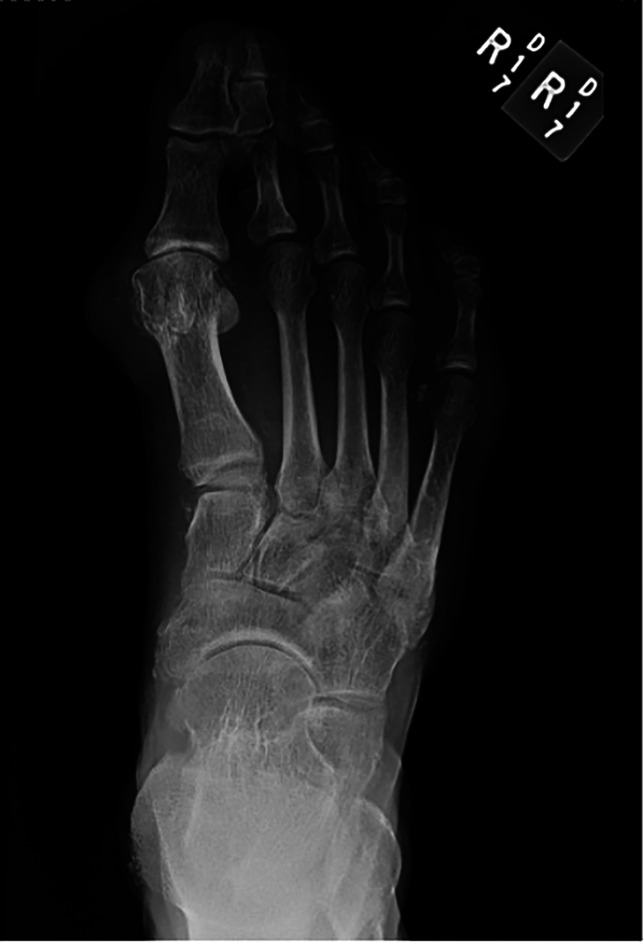
Radiographic imaging of gouty tophus associated with hallux valgus deformity showing joint destruction and medial soft tissue of tophus (author's clinical photograph).

**Figure 5:**
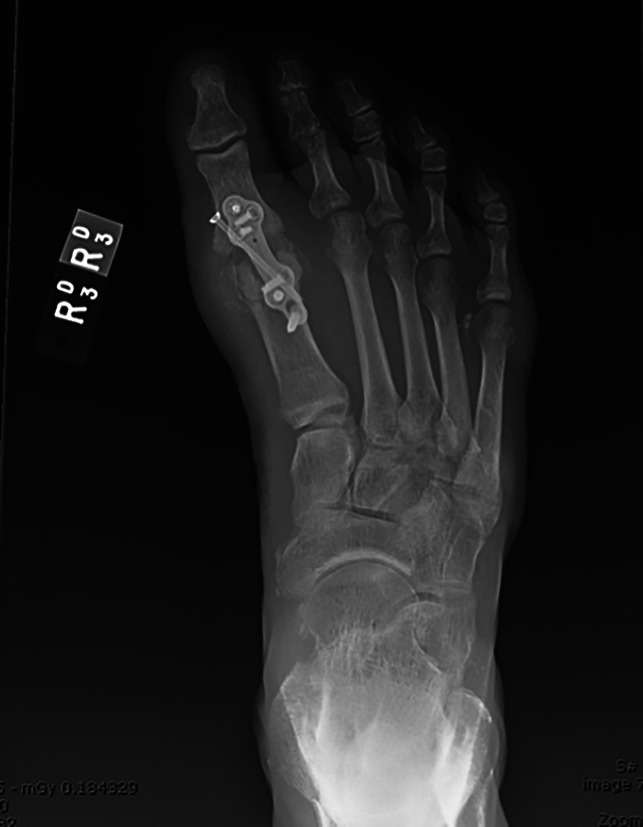
Postoperative radiographic imaging showing removal of tophi, first MTP joint deformity correction, and second PIP joint resection arthroplasty (author's clinical photograph). MTP = metatarsophalangeal, PIP = proximal interphalangeal

A case of a 58-year-old man with gouty first MTPJ arthritis and large associated tophus with severe clinical hallux valgus deformity is displayed in Figure 3. Interestingly, the bony hallux valgus deformity was minimal, and it was the presence of the large tophus that was the most clinically significant issue (Figure 4). This patient also had a painful tophus and deformity of the second toe. In this case, extensive soft-tissue débridement was performed along with a primary first MTPJ fusion and second toe PIP joint resection arthroplasty, as shown in Figure 5. This resulted in successful deformity correction and allowed the patient to return to wearing closed shoe wear.

Finally, there has been some research to suggest that arthroscopic irrigation and débridement of the first MTPJ in recalcitrant cases of gout can result in clinical improvement with reduced frequency of attacks; however, more work in this area is needed to determine which patients benefit from this approach.^40^

### Sesamoids

Several case reports have described gout as a potential cause of sesamoid pain, with all reported cases in younger adults aged 17 to 41 years.^41,42,43,44^ In these cases, gout was a diagnosis of exclusion and ultimately confirmed with histologic examination at the time of surgery. None of these patients reported a history of gout or had a preoperative elevated uric acid level. All cases were treated with either partial or complete excision of the affected sesamoid with resolution of symptoms. In two of those cases, the diagnosis of gout was in association with a procedure performed for a sesamoid fracture.^43,44^ Given that sesamoid pain can be difficult to treat nonsurgically, consideration to gout as a differential diagnosis is reasonable; however, based on the few reported cases, it is likely a rare etiology.^41^

### Tibialis Anterior

Gout has also been described as a rare cause of spontaneous tibialis anterior tendon rupture. When considering gout at a potential etiology for spontaneous tibialis anterior rupture, it is important to exclude other potential causes such as prior steroid injection into the tibialis anterior tendon sheath or systemic steroid treatment. Both nonsurgical treatment with ankle foot orthosis and surgical treatment with either direct repair of the tendon (with or without allograft augmentation) or a variety of tendon transfer techniques may be considered in these cases depending on the patients' functional goals and risk factors for surgery.^45^

### Other Sites

**Figure 6:**
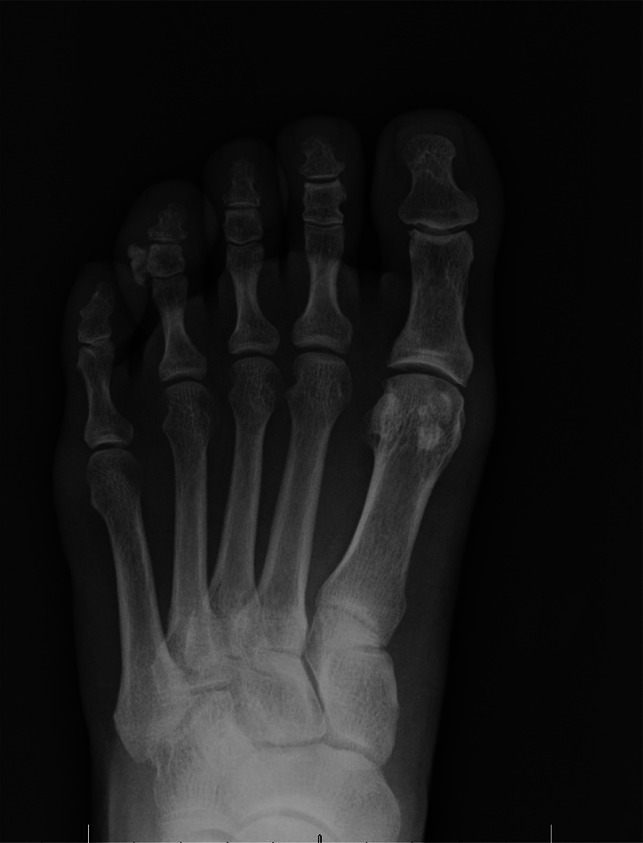
Radiograph depicting gouty tophus and deformity of the fourth toe middle phalanx (author's clinical photograph).

Gouty tophi have also been described in the tarsal bones and lesser toes, usually in the setting of prior first MTPJ involvement. One case describes successful surgical excision of a gouty tophus of the navicular, whereas other cases have been reported describing arthroscopic débridement with or without bone grafting for gout-related lesions of the talar dome.^46,47^ Figure 6 depicts an isolated gouty tophus of the fourth toe eventually treated with excision due to difficulty with shoe wear.

## Spine

The prevalence of gout in the spine remains unknown and is possibly underreported due to the invasive nature of obtaining tissue samples for diagnosis and the common coexistence of gout and osteoarthritis. Spinal gout can present acutely as back pain with or without neurologic deficits or chronically with symptoms developing over many years. Although gout in the spine usually presents in patients with a previous gout diagnosis, spinal involvement may be its initial presentation. A 2015 review of 131 case reports of gout with spinal involvement by Toprover at al.^48^ showed that as with acute gout flares in the knee, it is often difficult to exclude infection, given the common acute presentation of back pain, fever, and elevated inflammatory markers. In addition, MRI findings of gouty tophi in the spine may resemble osteomyelitis or tuberculosis. Diagnosis is generally made via a combination of advanced imaging (such as MRI with contrast, CT, and DECT) and image-guided biopsy or intraoperative tissue sample of the lesion.

Surgical intervention for gout in the spine should be considered when the gouty tophus is causing focal neurologic deficits or spinal cord compression.^48^ A 2019 case report by Akhter et al. describes a 26-year-old man with no known history of gout presenting with acute lower extremity numbness and weakness and multilevel thoracic lytic lesions on MRI/CT that was treated with urgent decompressive laminectomy, mass resection, and fusion. The authors noted a white, cheese-like material causing dural compression that was confirmed to have negatively birefringent crystals on analysis.^49^

Overall, gout with spine involvement should be considered in patients with a known history of gout and back pain that does not respond to conservative management. Diagnosis can be made with imaging via DECT and/or biopsy. Once the diagnosis is made, first-line treatment with urate lowering therapies is recommended, with surgical treatment to remove the gouty mass reserved for patients with spinal cord compression and focal neurologic deficits.^48^

## Summary

Gout is an increasingly common condition in the United States and often comorbid with hypertension, obesity, and chronic kidney disease. The benchmark for diagnosis is joint aspiration revealing uric acid crystals. Gout flares must be differentiated from septic arthritis and may be concurrently infected. The main treatment for acute and chronic gout is medical management of acute inflammation and chronic uric acid levels, but surgical treatment may be indicated in cases of damage to the surrounding soft tissue, concomitant septic arthritis, symptomatic cartilage loss, or neurologic deficits. In addition, gout does not seem to independently affect outcomes after total hip, knee, and ankle arthroplasty, but associated comorbidities should be considered. Finally, it is important for surgeons to collaborate with rheumatologists for perioperative management.

## Acknowledgment

We would like to acknowledge Cristina Terhoeve, MD, for her help in creating the manuscript proposal.
